# Italian Medical Professionals' Practices, Attitudes, and Knowledge in Travel Medicine: Protocol for a National Survey

**DOI:** 10.2196/59511

**Published:** 2025-04-21

**Authors:** Francesco Baglivo, Luigi De Angelis, Federico Vannini, Antonello Agostini, Antonio Todaro, Eleonora Torri, Giulio Alberto Gianolio Lopez, Margherita Fui, Alberto Tomasi, Caterina Rizzo

**Affiliations:** 1 Department of Translational Research and New Technologies in Medicine and Surgery University of Pisa Pisa Italy; 2 Italian Society of Travel Medicine and Migrations SIMVIM Livorno Italy

**Keywords:** travel medicine, Italy, cross-sectional, survey, KAP, medical professional, medical professionals, Italian, global health, epidemiology, scoping review, power analysis, dissemination, healthcare service, healthcare services, survey protocol, awareness, assessment

## Abstract

**Background:**

The evolving global health landscape highlights the importance of travel medicine, making it necessary for health care professionals to understand the epidemiologic profiles among varied traveler populations and keep themselves updated in this rapidly changing field. However, in Italy, travel medicine clinics have significant gaps in resource allocation, staff training, and infrastructure.

**Objective:**

This protocol of a cross-sectional study aims to create and validate a questionnaire to assess the knowledge, attitudes, and practices of health care professionals in travel medicine in Italy. The final goal is to provide a tool to evaluate the state of travel medicine, guide training initiatives, and be able to monitor trends over time.

**Methods:**

The study population consists of health care professionals who practice travel medicine in Italy. The questionnaire will be developed by adapting an existing English survey and conducting a scoping review to align the questionnaire with contemporary scientific discourse. The validation process includes face validity, content validity, and expert evaluation. The sample size, determined through power analysis, ranges from 218 to 278 participants. The questionnaire will undergo a pilot test on a smaller sample size (10% of the total) to identify and address any issues. Statistical analysis will include central tendency and dispersion measures, categorical summaries, group comparisons, and regressions. This research received ethical approval, and informed consent will be obtained from all participants.

**Results:**

As of July 2024, we completed the questionnaire validation involving 9 experts. The validated version of the questionnaire includes 86 items. Furthermore, we conducted a pilot test on 53 individuals during the SIMVIM (Italian Society of Travel Medicine and Migrations) course on travel medicine held in Lucca, Italy, on June 14, 2024.

**Conclusions:**

This cross-sectional study will guide strategic planning and targeting training and awareness activities in areas deemed most critical or lacking. The study’s structured approach and periodic assessments will facilitate the identification of educational gaps, the dissemination of best practices, and the overall improvement of health care services for travelers in Italy.

**International Registered Report Identifier (IRRID):**

DERR1-10.2196/59511

## Introduction

### Background

The ever-changing landscape of global health threats has made travel medicine an essential field for health care professionals. In the backdrop of dynamic global health scenarios, travelers play a significant role in the epidemiology of infectious diseases, as evidenced by the COVID-19 pandemic [1,2], as well as the spread of Ebola [3], Zika [4], and antimicrobial-resistant pathogens in recent years [5]. Their mobility, potential exposure to diseases outside their native countries, and the possibility of disease transmission across borders make them a crucial cohort for epidemiologic surveillance. By integrating international travelers into epidemiologic disease surveillance, it is possible to obtain a more nuanced understanding of the presence, frequency, seasonality, and geographic distribution [6]. This understanding might pivot over time due to various factors, such as outbreaks, climate change, vector habitat alterations, emerging or reemerging diseases, or effective public health initiatives [7-9].

It is necessary for health care professionals to understand the epidemiological profiles of the different types of travelers, keep themselves up-to-date in this rapidly evolving field, effectively inform travelers before departure, and offer posttrip assessments and possible treatments [10].

The quality of pretravel counseling, an indispensable measure for optimal risk management of travelers, hinges on the experience and knowledge of the health care professional providing the consultation [11]. It is through this expertise that personalized strategies to mitigate health risks—from behavioral norms to prescription prophylaxis and often expedited vaccination protocols—are crafted.

Around the European region, travel medicine is structured in highly different ways [12].

**Figure 1:**
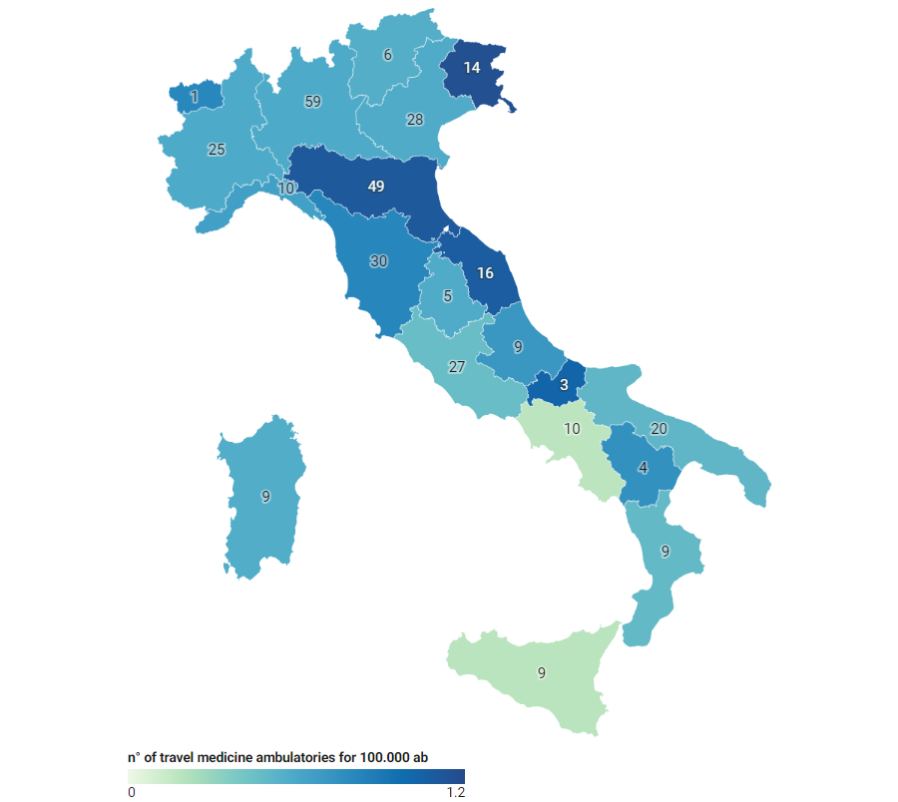
Number and density of travel medicine ambulatories in Italy.

In Italy, Local Health Units ASL (Azienda Sanitaria Locale, under the “Servizio sanitario nazionale,” SSN) have travel medicine specialized ambulatories that provide pre-travel medical consultations, vaccinations, and prophylaxis. These services are offered by the SSN in co-payment, resulting in cost savings for the travelers. In addition, the “Unità Sanitaria Marittima, Aerea e di Frontiera” (USMAF) at airports and ports offers travel medicine services. There are a total of 343 ambulatories; Table 1 and Figure 1 describe them in detail. Health care professionals working in these units can be doctors, nurses, and health assistants; they can provide information about health risks in different countries, mandatory or recommended vaccinations, and prophylaxis.

**Table 1:** Complete list of the authorized travel medicine ambulatories by the Italian Ministry of Health (decree 3 August 2022) by region. The population of that region in 2022 (Istituto Nazionale di Statistica [ISTAT]) and the density of ambulatories per 100,000 inhabitants are also listed.

Region	Travel medicine ambulatories, n	Population (2022 ISTAT), n	The density of ambulatories (n/100,000 ambulatories)
Abruzzo	9	1,269,860	0.71
Basilicata	4	536,659	0.75
Calabria	9	1,841,300	0.49
Campania	10	5,592,175	0.18
Emilia Romagna	49	4,426,929	1.11
Friuli Venezia Giulia	14	1,192,191	1.17
Lazio	27	5,707,112	0.47
Liguria	10	1,502,624	0.66
Lombardia	59	9,950,742	0.59
Marche	16	1,480,839	1.08
Molise	3	289,840	1.03
Piemonte	25	4,240,736	0.59
Puglia	20	3,900,852	0.51
Sardegna	9	1,575,028	0.57
Sicilia	9	4,802,016	0.19
Toscana	30	3,651,152	0.82
Umbria	5	854,137	0.58
Val d’Aosta	1	122,955	0.81
Veneto	28	4,838,253	0.58
Province Trento	2	542,050	0.37
Province Bolzano	4	533,267	0.75

Typical training is not required for travel medicine doctors working in these health facilities, even if many of them are infectious disease or public health specialists.

The Italian National Vaccination Plan (PNPV) for 2023-2025 highlights the importance of travel medicine [13]. It emphasizes that professionals should be well-informed about the global epidemiology of both infectious and noninfectious risks travelers face, current regulations, and immunization requirements in destination countries.

A national survey conducted in 2018 by the Italian Society of Travel Medicine and Migrations (SIMVIM) underlined the operational dynamics, challenges, and potential avenues for the evolution of Italian Travel Medicine Ambulatories [14].

A total of 79 travel medicine ambulatories participated in the survey nationwide. These clinics, on average, allocate about 10 hours per week to their services, corresponding to approximately 18 users each week. Concerning training, 70.9% of the health care staff had their last significant training or update in the 2-year window of 2017-2018. The survey showed that while these clinics play a commendable role in ensuring traveler health, considerable gaps exist in resource allocation, staff training, infrastructure, and integration with the broader National Health System.

Given this intricate background, there is an evident need for validated tools to assess the knowledge, attitudes, and practices (KAP) of health care professionals in the domain of travel medicine in Italy. These tools can offer a snapshot of the national scenario and potentially unveil a new path for health care professional training. In this context, we developed the protocol of IMPAKT (Italian Medical Professionals Practices, Attitudes and Knowledge in Travel Medicine), a cross-sectional study aimed at creating and validating a questionnaire to assess the KAP of health care professionals (doctors, nurses, and health assistants) involved in travel medicine within Italy. The survey will be disseminated through all available channels of SIMVIM and would be sent by email to official email addresses associated with travel medicine clinics.

### Objective of the Study

While the 2018 survey focused primarily on the operational aspects of travel medicine ambulatories, the aim of this study is to create and validate a questionnaire to assess the knowledge, attitudes, and practices of health care professionals in travel medicine in Italy, ultimately providing a tool to evaluate the state of travel medicine allowing a more detailed understanding of the educational and training needs at the individual and regional level in Italy.

## Methods

### Overview

**Figure 2:**
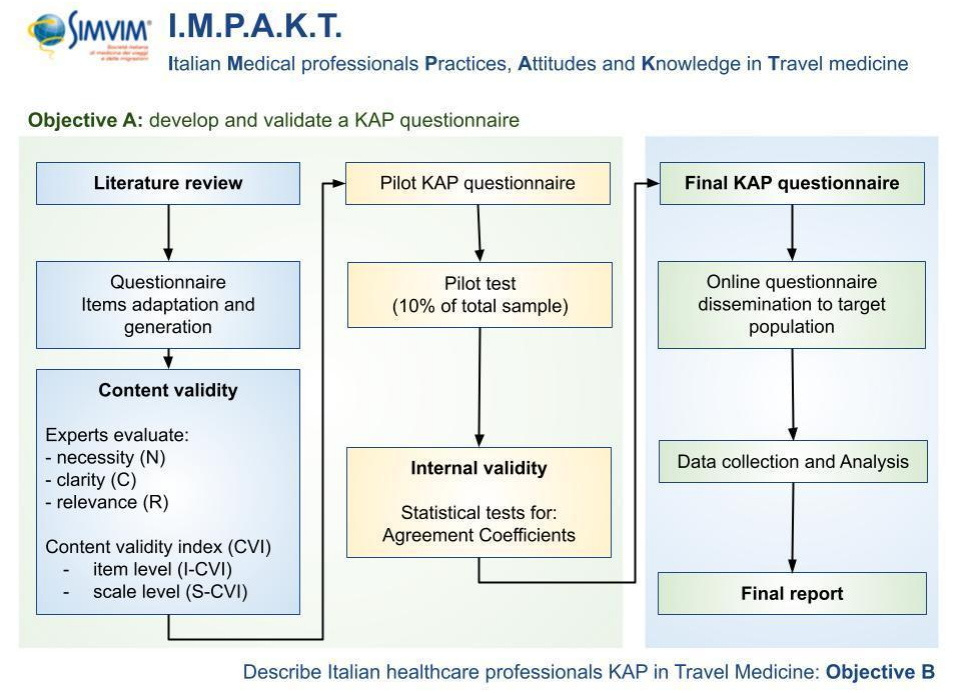
Diagram representing the phases and objectives of the Italian Medical Professionals Practices, Attitudes and Knowledge in Travel Medicine (IMPAKT) study. KAP: knowledge, attitudes, and practices.

KAP surveys are a widely used tool in cross-sectional studies to investigate health-related behaviors and health-seeking practices [15]. A KAP survey allows a target population to be studied in terms of knowledge, attitudes, and practices through the use of questionnaires (structured or semistructured) that are self-administered or administered by interviewers. Furthermore, the survey allows both qualitative and quantitative data to be collected [16]. Figure 2 shows a schematic representation of the proposed methodology.

The information for this study will be collected in a structured, self-administered questionnaire. For this study, “Knowledge in Travel Medicine” is defined as the understanding and awareness of health care professionals about travel-related health risks, prevention strategies, diagnostic measures, treatments, and appropriate interventions.

“Attitude toward Travel Medicine” among Italian health care professionals aims to analyze their perspectives, beliefs, feelings, and inclinations about the importance, relevance, and role of travel medicine in the patient’s general care. This involves assessing their perception of the value of travel health advice, perceived barriers, and their motivation to stay updated in this evolving field. “Practices of Travel Medicine” are defined by the actual behaviors, interventions, and actions taken by health care professionals involved in travel medicine. This includes the frequency and accuracy of pre-travel consultations, the administration of travel vaccinations, the provision of malaria and other travel-associated diseases prophylaxis, post-travel assessments, patient education on travel health precautions, and documentation practices, among others.

### Ethical Considerations

This research received ethical approval from the ethics committee of the University of Pisa on October 11, 2023 (review 39/2023; protocol 0136227/2023). Every participant will be provided with a privacy notice that explains the purpose of data collection, storage, and processing in compliance with the General Data Protection Regulation and Italian regulations. Before proceeding with the questionnaire, explicit consent will be obtained from the participants.

To ensure confidentiality and data protection, each participant will be assigned a unique identification code. The association between identifiers and emails will be kept in a separate file protected by a password. Data will be deleted 5 years after the end of the study. This procedure ensures that the data collected will be anonymized, safeguarding the privacy of all participants.

### Inclusion and Exclusion Criteria

The study population includes health care professionals (ie, doctors, nurses, or residents) who practice travel medicine in Italy. Textbox 1 shows the inclusion criteria for study participants.

**Inclusion and exclusion criteria.**

The following are the inclusion criteria:

- Being a health care professional (HCPs are medical doctors, nursing professionals, midwifery professionals, dentists, pharmacists, and trainees in the above categories).
- Practicing in Italy.
- Age≥18 years.
- Giving informed consent.

In addition, at least one of the following:

- Being a member of SIMVIM (or other associations or societies interested in travel medicine).
- Working or having worked in the last 5 years as a travel medicine consultant.
- Have participated in the previous 5 years in a workshop, seminar, or congress on travel medicine.
- Working in a travel medicine ambulatory.

The following are the exclusion criteria:

- Not being a health care professional.
- Practices the profession outside of Italy.
- Consent not given.
- Withdrawal of consent.

### Questionnaire Development

To structure the questionnaire for our study, we will adapt the one used by Kumar et al [17]. Kumar et al [17] developed a 106-item questionnaire in English to assess the existing expertise of Indian medicine and infectious diseases resident doctors with respect to travel medicine. We will adapt this English questionnaire to the Italian context, translating, modifying, and adding specific items to better capture the peculiarities of Italian travel medicine practice. Therefore, this already validated questionnaire will be a reference for the development of our questionnaire, as it has a similar aim and a similar target population.

Except for COVID-19–related papers, we will conduct a nonsystematic literature assessment of pertinent scientific publications over the last 5 years to guarantee a thorough representation of travel medicine issues in the questionnaire. This title-only review will cover a summary of the themes in travel medical literature with a title analysis. The aim is to align the questionnaire with the current scientific discourse on travel medicine. Multimedia Appendix 1 contains the PubMed query that will be used.

Regarding the translation of the Kumar et al [17] questionnaire, two separate translators among the investigators will translate each item (question) into Italian separately. The goal is to make sure there are no appreciable differences between the two translations and that they both express the same concept. A third translator will be sought in cases of disagreement. By evaluating the item and the two translations, this third translator will serve as an arbitrator, settling the conflict and producing a version that is agreed upon.

A back-translation will be carried out to confirm the accuracy of the translated items and to make sure the content has not been changed or misinterpreted during the translation process.

The English items and the back-translated English items will be compared to check for any discrepancies.

Items from the 2018 SIMVIM survey will be integrated into the questionnaire [14]. In addition, new items proposed by one investigator will undergo scrutiny by two other investigators before being added to the item pool to be examined by experts.

### Questionnaire Validation

In the following sections, the steps of the validation process of the questionnaire are described, those steps include the evaluation of face and content validity.

Face validity refers to experts’ initial agreement that a test is a legitimate representation of the domain in question [18]. After formulating the initial draft of our questionnaire, we will solicit feedback from experts in travel medicine. Their assessment is centered on readability, comprehensibility, feasibility, layout, and style, ensuring the questionnaire is valid in these aspects.

Content validity aims to minimize bias during the early stages of instrument operationalization [19].

To validate the content of our travel medicine questionnaire, we identified 3 key components to assess overall content validity. Indeed, we disassembled the overall validity into specific components [20], focusing on three determinants:

1. Necessity: Is the question necessary to be asked to the health care professional to assess their knowledge/attitude/practices of travel medicine? Each item can be rated as 1 (neither useful nor necessary), 2 (useful but not necessary), and 3 (essential).
2. Clarity: Are the question phrasing, structure, or provided options comprehensible? Each item can be rated as 1 (not clear), 2 (slightly clear/needs major revision), 3 (clear/needs minor revision), and 4 (very clear).
3. Relevance: Is the question pertinent to the domain of travel medicine, particularly in gauging knowledge, attitude, or practice? Each item can be rated as 1 (not relevant), 2 (slightly relevant/needs major revision), 3 (relevant/needs minor revision), and 4 (very relevant).

Experts will evaluate each question based on these determinants using a Likert scale. This approach allows specificity in content validation and facilitates a rigorous critique from experts. It also enables us to identify any discrepancies in expert opinions concerning the questionnaire's necessity, clarity, and relevance.

Experts (at least 3) with proven knowledge in travel medicine are selected to evaluate the content validity of the questionnaire. Experts enrolled in the study will provide their advice free of charge and sign informed consent to the study as other subjects.

At least one of these requisites is necessary to be qualified as an expert:

- A minimum of 10 years of clinical practice focused on travel medicine or related fields.
- Holding a Certificate in Travel Health (CTH) or similar certifications.
- Experience in educating or training other health care professionals about travel medicine, either as a faculty member of an educational institution or as a speaker in seminars/workshops.
- Contribution to the development of travel medicine guidelines or protocols.
- Authorship of peer-reviewed articles, book chapters, or research papers specifically focused on travel medicine or closely related fields.

An electronic questionnaire version will be shared with these experts alongside specific instructions about the validation objectives.

The item-level content validity index (I-CVI) and the scale-level content validity index (S-CVI) are the two main indices for determining a tool’s content validity; we will use both indexes, following other cross-sectional studies [20,21]. Our panel of experts will rate each item using the aforementioned criteria. Following this, scores will be dichotomized, converting them into “1” or “0” based on established rating criteria. Necessarily, the dichotomous variable will be categorized as “1” if the item is rated as “3” (essential) or “0” otherwise. Likewise, for clarity and relevance, the dichotomous variables will be categorized as “1” for experts, giving a rating of 3 or 4 and “0” otherwise.

Content validity indices will be computed (I-CVI and S-CVI/Ave [scale-level content validity index/average]) using these dichotomized scores, and the items with a score higher than the minimum required score (I-CVI≥0.82) will be included in the questionnaire. Polit and Beck have endorsed an S-CVI/Ave value of 0.90 or higher as excellent [22,23].

### Sample Size

The sample size for this cross-sectional study was calculated to determine the minimum number of travel medicine professionals needed to ensure a representative sample of the broader population of travel medicine professionals in Italy. Knowing that there are about 500 members affiliated with SIMVIM in 2022 and 343 authorized travel medicine ambulatories, we ran an analysis from 500 to 1000 as a possible population range. We set the confidence level at 95% and the margin of error at 5%, 8%, and 10%.

Based on these parameters and using the formula:




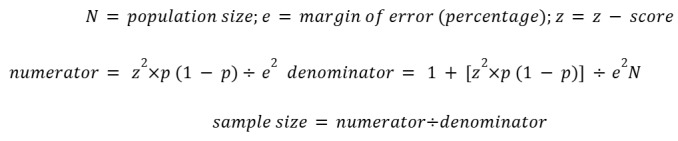




We determined the total sample sizes of respondents necessary to achieve sufficient statistical power, available in Table 2. Therefore, to achieve adequate statistical power, it is necessary to administer the questionnaire to a population sample of subjects between 218 and 278.

**Table 2:** Sample simulation, power analysis results.

Population size, n	Margin of error, %	Sample size, n
1000	5	278
500	5	218
1000	8	131
500	8	116
1000	10	88
500	10	81

### Pilot Test

A pilot cross-sectional study will be conducted on a smaller sample size (around 10% of the total sample size) to identify any issues with the expert-validated version of the questionnaire or data collection methods. The results collected from the pilot study will allow any adjustments to be made before the full-scale data collection.

The pilot study will be used to assess internal validity through agreement coefficients, which represent statistical measures used to quantify the degree of consensus or concordance among various respondents to a questionnaire. These parameters evaluate the consistency and reliability of ratings, taking into account the chance agreement. A Cronbach α over 0.7 will be considered sufficient, with a 0.8 benign optimal [24-26].

After evaluation of the aforementioned settings, we will finalize the KAP questionnaire, which will incorporate only items that meet the predetermined criteria.

### Dissemination and Data Collection

Upon validation and pilot testing, the questionnaire will be disseminated using an online form containing (1) an introduction to the questionnaire with the aim of the study, (2) a privacy disclaimer and informed consent, (3) a subject data section, and (4) a KAP questionnaire.

The form will be shared through all available channels of SIMVIM. As the primary association uniting professionals practicing travel medicine in Italy, SIMVIM provides an ideal platform for the widespread dissemination of the form. The study will be featured in SIMVIM’s regular email newsletter, ensuring it reaches all members directly. It will also be published on the official SIMVIM website, making it accessible to a broader audience. Furthermore, the study will be presented during in-person training and update events organized by SIMVIM for professionals in the field. Finally, the questionnaire link will be sent by email to official email addresses associated with travel medicine clinics. For each distribution method, we will track the number of individuals the questionnaire is presented to, enabling the calculation of a response rate. However, a potential limitation of our study is the overlap in membership between different contact methods, which may prevent the calculation of an overall response rate. For each participant, after giving the informed consent, the personal data in Table 3 will be collected through the online form, and an ID number will be assigned, which will substitute the email to anonymize results.

**Table 3:** Participant personal data to be collected in section 1 of the questionnaire.

Item (question)	Variable	Valid input	Reason
Q1	ID	Automatic (2023.001, 2023.002)	Anonymization
Q2	Date	Date (DD/MM/YYYY)	Participant identifier
Q3	Email	String @ string	Participant identifier
Q4	Age	Numeric (18-100)	Stratification and inclusion criteria
Q5	Sex	Factor (0=prefer not to say, 1=male, 2=female, and 3=other)	Stratification
Q6	Profession	Factor (0=medical doctor, 1=nurse, 2=health care assistant, 3=pharmacist, and 4=other health care professional)	Stratification and inclusion criteria
Q7	Work type	Factor (0=not working, 1=public fixed term, 2=public indeterminate, 3=private, 4=other, and 5=not working)	Stratification
Q8	Work region	Factor (21 levels, 19 regions, and 2 autonomous provinces)	Stratification
Q9	Country of practice	Factor (0=others, 1=Italy)	Stratification and inclusion criteria
Q10	Years of experience	Numeric (0-100)	Stratification and inclusion criteria
Q11	Travel medicine ambulatory	Factor (0=no and 1=yes)	Stratification and inclusion criteria
Q12	Education	Factor (high school diploma=1, university degree=2, graduate master=3, and PhD=4)	Stratification
Q13	Medical residency	Only for MD, string (description of the medical residency)	Stratification
Q14	Travel history	Numeric (number of intercontinental travels in the past 10 years)	Stratification
Q15	Main travel reason	Factor (0=job, 1=volunteering, 2=tourism, and 3=visiting friends and relatives)	Stratification
Q16	Workshop participation	Factor (0=no and 1=yes)	Stratification and inclusion criteria
Q17	Membership	Factor, more than one possible (0=none, 1=SIMVIM, 2=SITI, and 3=other)	Stratification and inclusion criteria

### Statistical Analysis

The data obtained from the final questionnaire will undergo statistical analysis to ensure a comprehensive interpretation. The measurements of the central trend (mean and median) and dispersion (SD and IQR), depending on the part of the questionnaire analyzed, will be calculated. Categorical responses will be summarized using percentages, frequencies, and graphical representations (bar charts, histograms, and pie charts) will be used for visual summaries. Differences among groups in KAP scores will be assessed using *t* tests (2 groups) or ANOVA (2+ groups). Mann-Whitney *U* test or Kruskal-Wallis test will be used if the data does not meet the assumptions of the parametric tests. Chi-square tests or Fisher exact tests will be applied to compare categorical data among groups.

When assessing predictors of a particular outcome (eg, certain knowledge scores predicting practices), either multiple linear regression (for continuous outcomes) or logistic regression (for binary outcomes) will be used. Relevant assumptions for each regression model will be verified before the analysis is performed.

A *P* value of less than <.05 will be considered statistically significant for all tests. When conducting multiple comparisons, correction methods such as Bonferroni correction will be applied to check the familywise error rate. All statistical analyses will be performed using the statistical software R (R Foundation for Statistical Computing).

To ensure geographical representation, considering that the Italian health care system is organized federally, the stratification of results by region will be conducted only if a significant proportion of respondents declare to operate in that region (Q8). If not all the 19 regions and 2 autonomous provinces are sufficiently represented, an additional stratification by macro area will be conducted.

## Results

The PubMed query described in the Methods section yielded a total of 1067 articles published between 2018 and 2023. A screen of the article titles allowed us to identify 13 overarching topics into which knowledge items could be classified. The identification of these topics was primarily guided by the structure of the CDC Yellow Book [27], which provides a comprehensive framework for travel medicine. The literature retrieved through PubMed was subsequently used to corroborate the relevance and coverage of the selected topics. The following topics represent key domains in travel medicine: (1) general principles of travel medicine, (2) pretravel consultation, (3) posttravel consultation, (4) vaccination and immunoprophylaxis—general principles, (5) travelers’ diarrhea, (6) travel-associated infections including malaria, (7) yellow fever, (8) bacterial diseases, (9) viral diseases, (10) travelers with additional considerations, (11) travel by air, land, and sea, (12) environmental hazards and risks, and (13) travel medicine ambulatory organization.

As of July 2024, we completed the questionnaire validation involving 9 experts. The validated version of the questionnaire includes 86 items. Furthermore, we conducted a pilot test on 53 individuals during the SIMVIM course on travel medicine held in Lucca, Italy, on June 14, 2024.

## Discussion

This research project, jointly developed by the University of Pisa and the SIMVIM, aims to create a “diagnostic” tool for assessing the state of Travel Medicine in Italy. Given the inherent nature of travel medicine, as also highlighted in the PNPV 23-25 [13], it is crucial to ensure that health care professionals providing travel medicine services are well-trained and continuously updated to deliver high-level services to travelers.

This cross-sectional study, as outlined in the protocol, will serve as a guide to strategically plan and target training and awareness activities in areas deemed most critical or lacking. These activities are already being conducted by SIMVIM and other scientific societies focused on travel medicine in Italy, but without a structured approach due to the lack of a national standardized evaluation tool. In addition, by periodically repeating the questionnaire (annually or biennially), it will be possible to monitor the trends in knowledge, practices, and attitudes of health care professionals, thus ecologically assessing the impact of organized activities in Italy.

Specifically, it will be feasible to evaluate whether the level of knowledge on each topic has increased or decreased based on the themes covered in “update workshops” and conferences organized nationally. This approach will also consider the equity principle at the base of the Italian national health system [28], identifying any educational gaps in specific geographical areas of the country to make it possible to address them with specific interventions.

Furthermore, in the spirit of equity and equal access to care and prevention, systematic collection and analysis of practices used in travel medicine clinics will be conducted. This will help assess the national status and identify best practices to be disseminated nationally. If any shortcomings or structural deficiencies are highlighted, solutions could be formulated with the involvement of stakeholders, whether political or from the industry.

In conclusion, we believe that the effort to develop a tool refined by national experts can significantly contribute to the development and dissemination of good practices and better knowledge of travel medicine in Italy. Sharing this protocol could also lead other Beveridge health care systems to replicate a similar approach, thereby enhancing prevention and care to safeguard the health of travelers.

## Appendix

Multimedia Appendix 1 Additional material.

## Abbreviations

ASL: Azienda Sanitaria Locale
CTH: Certificate in Travel Health
I-CVI: item-level content validity index
IMPAKT: Italian Medical Professionals Practices, Attitudes and Knowledge in Travel Medicine
KAP: knowledge, attitudes, and practices
PNPV: Italian National Vaccination Plan
S-CVI/Ave: scale-level content validity index/average
S-CVI: scale-level content validity index
SIMVIM: Italian Society of Travel Medicine and Migrations
USMAF: Unità Sanitaria Marittima, Aerea e di Frontiera

## Data Availability

The data sets generated during and/or analyzed during this study are not publicly available due to privacy restrictions.

## References

[1] Bauer IL (2022). COVID-19: how can travel medicine benefit from tourism's focus on people during a pandemic?. Trop Dis Travel Med Vaccines.

[2] Flaherty GT, Hamer DH, Chen LH (2022). Travel in the time of COVID: a review of international travel health in a global pandemic. Curr Infect Dis Rep.

[3] Cardona-Ospina JA, Giselle-Badillo A, Calvache-Benavides CE, Rodriguez-Morales AJ (2014). Ebola virus disease: an emerging zoonosis with importance for travel medicine. Travel Med Infect Dis.

[4] Zika in travellers 1947–2017: a systematic review | Journal of Travel Medicine | Oxford Academic [Internet].

[5] Bokhary H, Pangesti KNA, Rashid H, Abd El Ghany M, Hill-Cawthorne GA (2021). Travel-related antimicrobial resistance: a systematic review. Trop Med Infect Dis.

[6] Rolka H, Walker DW, English R, Katzoff MJ, Scogin G, Neuhaus E, Centers for Disease ControlPrevention (2012). Analytical challenges for emerging public health surveillance. MMWR Suppl.

[7] Fisman DN (2007). Seasonality of infectious diseases. Annu Rev Public Health.

[8] Lambin EF, Tran A, Vanwambeke SO, Linard C, Soti V (2010). Pathogenic landscapes: interactions between land, people, disease vectors, and their animal hosts. Int J Health Geogr.

[9] Carmona P, Gandon S (2020). Winter is coming: Pathogen emergence in seasonal environments. PLoS Comput Biol.

[10] Zimmer R (2012). The pre-travel visit should start with a "risk conversation". J Travel Med.

[11] Murray HW (2020). The pretravel consultation: recent updates. Am J Med.

[12] Schlagenhauf P, Santos-O'Connor F, Parola P (2010). The practice of travel medicine in Europe. Clin Microbiol Infect.

[13] EpiCentro. Piano nazionale di prevenzione vaccinale (PNPV) 2023-2025 [Internet].

[14] Canale A, Giardi F, Giorgi S, Lopalco P, Tomasi A (2020). Travel Clinics, where we are and where we are going: a national survey in Italy. European Journal of Public Health.

[15] Kaur N, Haloi R, Ingle NA (2014). KAP surveys and oral health: a detailed review. Journal of Contemporary Dentistry Aug.

[16] (2014). SPRING. SPRING.

[17] Kumar A, Rajendran A, Usman M, Ahuja J, Samad S, Mittal A, Garg P, Baitha U, Ranjan P, Wig N (2022). Development and validation of a questionnaire to evaluate the knowledge, attitude and practices regarding travel medicine amongst physicians in an apex tertiary hospital in Northern India. Trop Dis Travel Med Vaccines.

[18] Frantz A, Holmgren K (2019). The work stress questionnaire (WSQ) - reliability and face validity among male workers. BMC Public Health.

[19] Taherdoost H (2016). Validity and reliability of the research instrument; how to test the validation of a questionnaire/survey in a research. SSRN Journal.

[20] Halek M, Holle D, Bartholomeyczik S (2017). Development and evaluation of the content validity, practicability and feasibility of the innovative dementia-oriented assessment system for challenging behaviour in residents with dementia. BMC Health Serv Res.

[21] Rodrigues IB, Adachi JD, Beattie KA, MacDermid JC (2017). Development and validation of a new tool to measure the facilitators, barriers and preferences to exercise in people with osteoporosis. BMC Musculoskelet Disord.

[22] Polit DF, Beck CT (2006). The content validity index: are you sure you know what's being reported? Critique and recommendations. Res Nurs Health.

[23] Kovacic D (2017). Using the content validity index to determine content validity of an instrument assessing health care providers’ general knowledge of human trafficking. Journal of Human Trafficking.

[24] (2018). Implementing a General Framework for Assessing Interrater Agreement in Stata - Daniel Klein, 2018 [Internet].

[25] Nyarko NY, Addo H (2013). Effects of teachers level of education and experience on teacher-child interactions in early childhood institutions. PSYCH.

[26] de Raadt A, Warrens MJ, Bosker RJ, Kiers HAL (2021). A Comparison of reliability coefficients for ordinal rating scales. J Classif.

[27] Nemhauser J, Rocque RL, Alvarado-Ramy F, Angelo K, Ericsson C, Gertz A, Kozarsky P, Ostroff S, Ryan E, Shlim D, Nemhause JB (2024). CDC Yellow Book 2024: Health Information for International Travel.

[28] Masseria C, Giannoni M (2010). Equity in access to health care in Italy: a disease-based approach. Eur J Public Health.

