# Supplementary material for: Italian Medical Professionals' Practices, Attitudes, and Knowledge in Travel Medicine: Protocol for a National Survey
Source: JMIR Res Protoc. 2025 Apr 21;14:e59511. doi: 10.2196/59511 (PMC12053279; doi:10.2196/59511)
Supplement: Multimedia Appendix 1 [file resprot_v14i1e59511_app1.docx]

**Supplementary materials**

S1: Query used for the scoping review on PubMed:

("travel medicine"[MeSH Terms] OR ("travel medicine"[MeSH Terms] OR ("travel"[All Fields] AND "medicine"[All Fields]) OR "travel medicine"[All Fields]) OR (("travel"[MeSH Terms] OR "travel"[All Fields] OR "traveling"[All Fields] OR "travelling"[All Fields] OR "travels"[All Fields] OR "traveled"[All Fields] OR "traveler"[All Fields] OR "traveler s"[All Fields] OR "travelers"[All Fields] OR "travelled"[All Fields] OR "traveller"[All Fields] OR "traveller s"[All Fields] OR "travellers"[All Fields]) AND ("health"[MeSH Terms] OR "health"[All Fields] OR "health s"[All Fields] OR "healthful"[All Fields] OR "healthfulness"[All Fields] OR "healths"[All Fields])) OR (("travel"[MeSH Terms] OR "travel"[All Fields] OR "traveling"[All Fields] OR "travelling"[All Fields] OR "travels"[All Fields] OR "traveled"[All Fields] OR "traveler"[All Fields] OR "traveler s"[All Fields] OR "travelers"[All Fields] OR "travelled"[All Fields] OR "traveller"[All Fields] OR "traveller s"[All Fields] OR "travellers"[All Fields]) AND "vaccin*"[All Fields]) OR ("travel-related"[All Fields] AND "illness*"[All Fields]) OR ("pre-travel"[All Fields] AND ("advice"[All Fields] OR "advices"[All Fields])) OR ("post-travel"[All Fields] AND "disease*"[All Fields])) AND (2018:2023[pdat]) NOT COVID-19[MeSH Terms]
